# Differential Gene Repertoire in *Mycobacterium ulcerans* Identifies Candidate Genes for Patho-Adaptation

**DOI:** 10.1371/journal.pntd.0000353

**Published:** 2008-12-23

**Authors:** Michael Käser, Gerd Pluschke

**Affiliations:** Swiss Tropical Institute, Basel, Switzerland; University of Tennessee, United States of America

## Abstract

**Background:**

Based on large genomic sequence polymorphisms, several haplotypes belonging to two major lineages of the human pathogen *Mycobacterium ulcerans* could be distinguished among patient isolates from various geographic origins. However, the biological relevance of insertional/deletional diversity is not understood.

**Methodology:**

Using comparative genomics, we have investigated the genes located in regions of difference recently identified by DNA microarray based hybridisation analysis. The analysed regions of difference comprise ∼7% of the entire *M. ulcerans* genome.

**Principal Findings:**

Several different mechanisms leading to loss of functional genes were identified, ranging from pseudogenization, caused by frame shift mutations or mobile genetic element interspersing, to large sequence polymorphisms. Four hot spot regions for genetic instability were unveiled. Altogether, 229 coding sequences were found to be differentially inactivated, constituting a repertoire of coding sequence variation in the rather monomorphic *M. ulcerans*.

**Conclusions/Significance:**

The differential gene inactivation patterns associated with the *M. ulcerans* haplotypes identified candidate genes that may confer enhanced adaptation upon ablation of expression. A number of gene conversions confined to the classical lineage may contribute to particular virulence of this group comprising isolates from Africa and Australia. Identification of this spectrum of anti-virulence gene candidates expands our understanding of the pathogenicity and ecology of the emerging infectious disease Buruli ulcer.

## Author Summary

The emerging human disease Buruli ulcer, caused by *Mycobacterium ulcerans*, is of increasing challenge for public health systems in many countries, mainly in West and Central sub-Saharan Africa. Genetic differentiation of patient isolates, a prerequisite for scientific studies on and intervention of disease transmission and dispersal, is hampered by an exceptional lack of genetic diversity within this species. Comparative genomics on *M. ulcerans* of worldwide geographical origin has already allowed for distinguishing several haplotypes separated into two distinct lineages. Differences in prevalence and incidence of Buruli ulcer were already suspected, but biological relevance for this was unclear. Here, we show newly identified hot spot regions of genomic instability, a biased silencing of coding sequences belonging to distinct functional groups, and a differential gene repertoire across *M. ulcerans* strains. Gene inactivation mediated by different mechanisms in *M. ulcerans* adds to the concept of anti-virulence genes observed in an increasing number of bacterial species. According to this concept, loss of such genes—in addition to gain of function—may confer a selective advantage for a pathogen radiating into a new niche. In the case of *M. ulcerans*, a distinct set of disrupted genes may enhance virulence, particularly in the classical lineage.

## Introduction

*Mycobacterium ulcerans* is the etiologic agent of the emerging human disease Buruli ulcer, the third most common mycobacterial disease which occurs in more than 30 countries. It is associated with necrosis of subcutaneous tissues, mainly in the extremities of children, and often leads to severe disability. Due to an exceptional lack of genetic diversity in *M. ulcerans* genetic fingerprinting methods for studies on disease transmission are currently not available [1]–[4]. In *M. tuberculosis*, single nucleotide polymorphisms (SNPs) and large sequence polymorphisms (LSPs) are used to investigate global dissemination and to rapidly track transmission pathways [5]–[7]. Earlier, we have identified regions of difference (RDs) between *M. ulcerans* patient isolates originating from different geographical areas [8]. These genomic variations caused by deletions, combined insertions/deletions (InDels), insertions of mobile insertion sequence elements (ISEs), and genome rearrangements proved useful genetic markers for phylogenetic analyses [9]. There is evidence that the most recent common ancestor of *M. ulcerans* has developed from the fish pathogen *M. marinum* [9]–[11] for which a whole genome sequence was recently completed [12]. We have identified six InDel haplotypes that can be grouped into two distinct lineages: the ancestral lineage comprising the haplotypes from Asia, South America, and Mexico, that is genetically closer to *M. marinum* in RD composition, and the classical lineage comprising the haplotypes originating from Africa, Australia, and South East Asia [9],[13]. Although the number of Buruli ulcer cases may be largely underestimated in some of the endemic countries, the main prevalence is in West-Africa [14]. The continental distribution of severe disease focussing on West-Africa and Australia correlates with the presence of the *M. ulcerans* classical lineage, which is increasingly suspected to be more pathogenic than the ancestral lineage [9],[15],[16].

The major virulence determinant of *M. ulcerans* is the immunosuppressive and cytotoxic macrolide toxin, mycolactone, produced by enzymes encoded by the virulence plasmid, pMUM001 [17],[18]. In addition to such gain-of-function pathogenic factors, virulence can also be determined by genes that confer enhanced adaptation upon loss of their function, since their expression is detrimental for a pathogen radiating into new niches. Such factors, designated anti-virulence genes [19],[20], are being identified for an increasing number of prokaryotic pathogens (e.g. [21]–[25]) including *M. tuberculosis* [26]–[29]. Orthologues of CDSs that are essential for pathogenicity in *M. tuberculosis*, such as members of the ESX-1 secretion apparatus and α-crystallin-like protein (HspX), were recently shown to be differentially affected by gene inactivation between the haplotypes of *M. ulcerans*, probably for reasons of evasion from the hosts' immune system [13].

In this report, we provide a detailed description of RDs among the otherwise genetically monomorphic *M. ulcerans* patient isolates of world-wide origin, covering ∼7% of the whole genome and comprising 338 coding sequences (CDSs). First, this comprehensive comparison led to the identification of a set of genes that were differentially inactivated across *M. ulcerans* haplotypes. Second, this differential gene repertoire may have implications for lineage specific differences in ecology and virulence of *M. ulcerans* and the predominant prevalence of Buruli ulcer in West-Africa and Australia. We hypothesize that, in addition to the acquisition of the plasmid, comprising the mycolactone encoding gene cluster, loss of distinct anti-virulence genes was important for the development of a highly virulent lineage of mycolactone producing mycobacteria.

## Materials and Methods

### Mycobacterial strains

*M. ulcerans* strains isolated from lesions of human Buruli ulcer patients used in this study are as follows (for a more detailed description see [8]). For the classical lineage: Ghana IFIK 1066089 (this study), Ghana Agy99, Ghana ITM 970321, Ghana ITM 970359, Ghana ITM 970483, Ivory Coast ITM 940662, Ivory Coast ITM 940815, Ivory Coast ITM 940511, Benin ITM 970111, Benin ITM 940886, Benin ITM 940512, Benin ITM 970104, Democratic Republic of Congo (DRC) ITM 5150, DRC ITM 5151, DRC ITM 5155, Togo ITM 970680, Angola ITM 960657, Angola ITM 960658, Papua New Guinea (PNG) ITM 941331, PNG ITM 9537, Malaysia ITM 941328, Australia ITM 941324, Australia ITM 941325, Australia ITM 941327, Australia ITM 9549, Australia ITM 9550, Australia ITM 8849, Australia ITM 940339, Australia ITM 5142, and Australia ITM 5147. For the ancestral lineage: China ITM 980912, Japan ITM 8756, French Guiana ITM 7922, Surinam ITM 842, Mexico ITM 5114, and Mexico ITM 5143. The clinical isolate *M. marinum* strain M (ATCC BAA-535) was used for interspecies comparison.

### Genomic DNA extraction

Bacterial pellets of about 60 mg (wet weight) were heat inactivated for 1 hour at 95°C in 500 µl extraction buffer (50 mM Tris-HCl, 25 mM EDTA, 5% monosodium glutamate), and sequentially treated with lysozyme (2 h, 37°C, 17 M lysozyme) and proteinase K (overnight, 45°C, 0.3 M proteinase K in proteinase K buffer: 1 mM Tris-HCl, 5 mM EDTA, 0.05% SDS, pH7.8). After digestion, the samples were subjected to bead beater treatment (7 min, 3000 rpm, Mikro-Dismembrator, B. Braun Biotech International, Melsungen, Germany) with 300 µl of 0.1 mm zirconia beads (BioSpec Products, Bartlesville, OK, USA). DNA was extracted from the supernatants by phenol-chloroform (Fluka, Buchs, Switzerland) extraction and subjected to ethanol precipitation. DNA concentration was measured by optical density at 260 nm (GeneQuant spectrophotometer, Pharmacia Biotech, Cambridge, UK).

### DNA amplification and sequencing

PCR was performed using FirePol 10× BD buffer and 0.5 µl FirePolTaq-Polymerase (Solis BioDyne, Tartu, Estonia), 5 ng genomic DNA or the corresponding volume of RNAse free water as a negative control, 0.6 µM forward and reverse primers each, 1.7 mM MgCl_2_ and 0.3 mM of each dNTP in a total volume of 30 µl. Long-range PCR polymerase mix (Fermentas, St. Leon-Rot, Germany) was applied according to the manufacturer's protocol to retrieve PCR products longer than 3 kb and up to 8 kb. PCR reactions were run in a GeneAmp PCR System 9700 PCR machine. The thermal profile for PCR amplification of *M. ulcerans* genomic DNA included an initial denaturation step of 95–98°C for 3 min, followed by 32 cycles of 95°C for 20 sec, annealing at 58–65°C for 20 sec, and elongation at 72°C for 30 sec up to 4 min. The PCR reactions were finalized by an extension step at 72°C for 10 min. PCR products were analyzed on 1–2% agarose gels by gel electrophoresis using ethidium bromide staining and the AlphaImager illuminator (Alpha Innotech, San Leandro, CA, USA). PCRs fragments produced for analysis of unknown genomic sequences were purified using the NucleoSpin purification kit (Machery-Nagel, Düren, Germany) and subjected to direct sequencing or cloned using the TOPO TA Cloning Kit (Invitrogen Corporation, Carlsbad, CA, USA), transformed into JM109 (Sigma-Aldrich, Buchs, Switzerland) bacterial cells, and sequenced after DNA preparation (Miniprep-Kit, Sigma-Aldrich, Buchs, Switzerland). Sequencing was performed using the Big Dye kit and the AbiPrism310 genetic sequence analyzer (Perkin-Elmer, Waltham, MA, USA). Primers (Sigma-Aldrich, Steinheim, Germany) were selected on the genome sequences of *M. ulcerans* Agy99 (Genbank accession number CP000325) and *M. marinum* M (Genbank accession number CP000854 and CP000895) using the Primer3 software (http://frodo.wi.mit.edu/cgi-bin/primer3/primer3_www.cgi) and, for unknown regions, combined with outward directed primers corresponding to sequences within the IS*2404* and IS*2606* elements.

### Real-time PCR

Primers (Sigma-Aldrich, Steinheim, Germany) and TaqMan probes (Biomers, Ulm, Germany) were designed using the Primer Express software version 2.0 (Applied Biosystems, Foster City, CA, USA), probes were 5′ labeled each with fluorescent dye, FAM, and 3′ labeled with the quencher, TAMRA. Primers and probes targeted *M. ulcerans* Agy99 sequences of IS*2404* (IS*2404*cf AAAGCACCACGCAGCATCTT, IS*2404*cr AGCGACCCCAGTGGATTG, and IS2404cp FAM-CCGTCCAACGCGATCGGCA-TAMRA), IS*2606* (IS*2606*f TGCTGACGGAGTTGAAAAACC, IS*2606*r CCTTTGAGGCCGTCACAGA, and IS*2606*p FAM-CGGCGTGGCCGACATCTTCTTC-TAMRA), and GroEL (GroELf CCTGCTGAGCGTCGAAGTC, GroELr GGGCACCGAGCTGGAGTT, and GroELp FAM-CCGAGAGGTATCCCTTGTCGAAACCG-TAMRA). Real-time PCR mixtures contained 50 fg of template DNA, 900 nM of TaqMan probe and 300 nM of each primer, and TaqMan Universal PCR Master Mix (Applied Biosystems, Foster City, CA, USA) in a total volume of 25 µl. Amplification and signal detection were performed using the 7500 Real Time PCR System (Applied Biosystems, Foster City, CA, USA) at the following conditions: 1 cycle of 50°C for 2 min, 1 cycle of 95°C for 10 min, 40 cycles of 95°C for 15 s and 60°C for 1 min. Quantitative TaqMan real-time PCR CT values for the ISEs were normalized by detection of the single copy GroEL target sequence. Samples were repeated at least twice and negative controls were included in each assay. The estimated difference in mean CT values between the lineages was calculated together with the 95% confidence interval (CI).

### DNA sequence analysis and bioinformatics

For four InDel haplotypes the following strains were used as representatives: Ghana IFIK 1066089 and Ghana 970359; Australia 941324 and Australia 940339; China 980912 and Japan 8756; French Guyana 7922 and Surinam 842; Mexico 5143 and Mexico 5114 [9]. The two haplotypes within the classical lineage, Australia 5142/47 and Australia 9549 [8],[13], differed only in one InDel each from Agy99 and thus were excluded from the RD description. DNA sequences were retrieved using a combination of genome sequence scanning, primer walking, and sequence gap bridging, as described earlier [9]. Sequences were aligned to the recently published *M. marinum* M genome [12] for absence or presence of CDSs. Comparative *in silico* sequence analysis was performed using the sequence manipulation suite (http://bioinformatics.org/sms/index.html), the sequence alignment tool blast 2 sequences (http://www.ncbi.nlm.nih.gov/blast/bl2seq/wblast2.cgi), the multiple sequence alignment website Multalin (http://bioinfo.genopole-toulouse.prd.fr/multalin/multalin.html), the Artemis software release 9 [30], and the Artemis Comparison Tool software release 6 [31].

## Results

**Figure 1:**
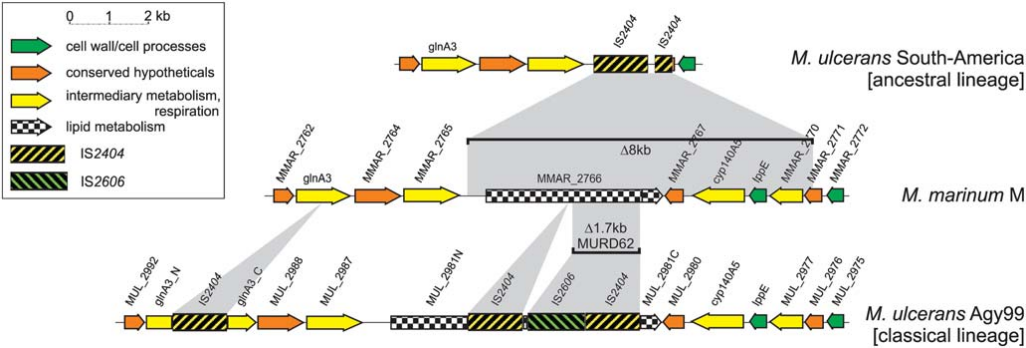
Comparison of CDSs (coding sequences) in RD (region of difference) 1 between *M. marinum* M (centre line), *M. ulcerans* haplotype South America (upper line, a member of the ancestral lineage) and *M. ulcerans* Agy99 (bottom line, a member of the classical lineage). Deletions (bars) and insertions as compared to the *M. marinum* M sequence are indicated by grey areas. Note that “MURDs” (*M. ulcerans* regions of difference) only define differences between *M. marinum* M and *M. ulcerans* Agy99, member of the classical lineage. Therefore, regions varying between *M. ulcerans* strains should be described as “RDs” (region of differences).

**Figure 2:**
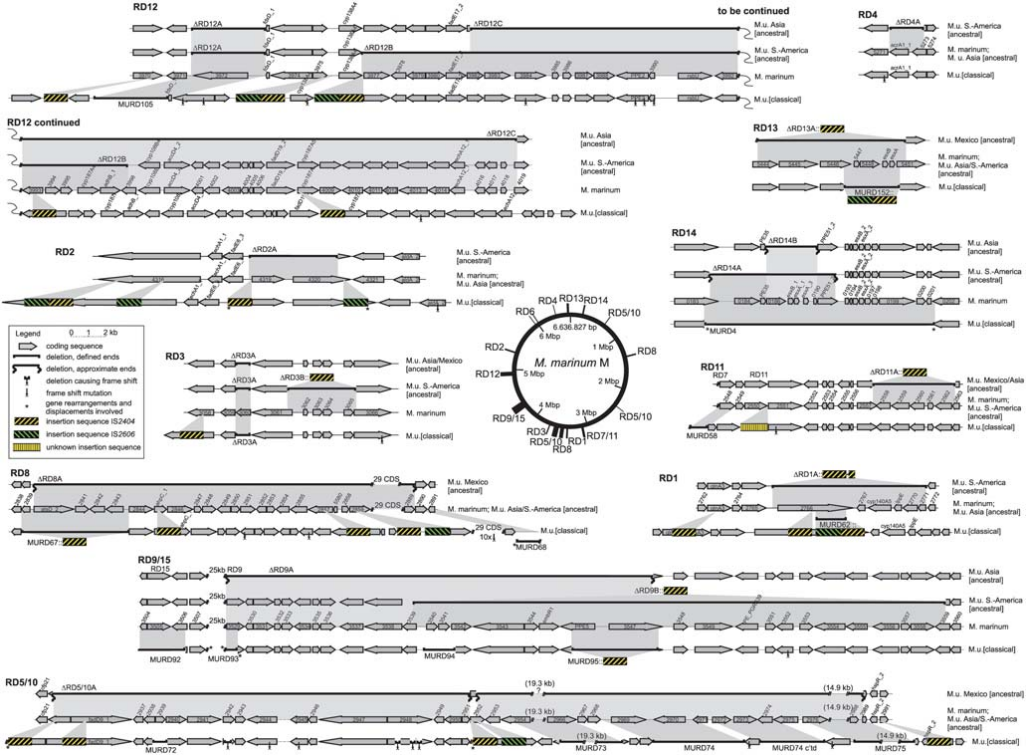
Description of RDs1-15 throughout the *M. ulcerans* haplotypes. Shown are the CDSs of the *M. marinum* M sequence backbone (which is closest to the entirety of all *M. ulcerans* haplotypes and thus to an *M. ulcerans* most recent common ancestor). Variations thereof in *M. ulcerans* haplotypes are depicted above the *M. marinum* sequence for members of the ancestral lineage, as indicated, and below for the classical lineage (exemplified by Agy99). Grey areas indicate differences of insertions, deletions, or InDels, as compared to *M. marinum*. Nomenclature of CDSs is indicated along the *M. marinum* annotation [40], i.e. 3970 stands for MMAR_3970. Symbol explanations see legend. Note that some genomic loci of Agy99 can neither be aligned directly to *M. marinum* M nor to *M. ulcerans* ancestral haplotypes due to major sequence rearrangements and displacements in the classical lineage only (indicated with *). Pseudogenes caused by frame shift mutations, according to the *M. ulcerans* Agy99 genome annotation, are marked as “x”. Bar = deletion. Blunt ends of bars: breakpoints exactly defined. Blurred ends of bars: breakpoints approximately. MURDs are confined to the *M. ulcerans* classical lineage but are usually not deleted in the ancestral lineage. Not shown are RDs6 and 7 since they do not reveal deletions relative to *M. marinum* M. A corresponding and complete list of silenced CDS is supplied in Table S1.

Within the analysed 7% of the entire *M. ulcerans* genome associated with RDs1 to 15 we observed various genetic mechanisms that led to specific ablation of the expression of sets of proteins across the six haplotypes: i) frameshift mutations resulting in pseudogenization, ii) interspersing of ISEs into CDS that led to their disruption, and iii) physical deletions of sizes between 2 and 53 kbp with replacement by ISEs which made their involvement obvious. Both pseudogenization or functional disruption, leaving the CDSs with scars in the genome, and physical deletion of the CDSs lead to gene silencing. Throughout the RDs, there is a strong bias of the two *M. ulcerans* lineages in their mechanisms leading to gene loss [9]: in the ancestral lineage deletions of large DNA stretches play a major role, whereas the classical lineage shows preponderance of ISEs interrupting CDSs, often even without concurrent deletions, as shown for RD1 in Fig. 1. Although a sequence of events cannot be deduced for RD1 from Fig. 1, it is clear that the inactivation of MMAR_2766, involved in lipid metabolism, was mediated by independent InDel events in the two lineages. In the ancestral lineage, five additional genes were lost with the 8 kb deletion whereas only in the classical lineage interspersing of an IS*2404* element into glnA3 led to its functional disruption (Fig. 1). Thus, independent InDel events have led to a differential gene repertoire between the two lineages. Fig. 2 gives a comprehensive reference overview of all genome variations in the identified RDs1 through 15 and shows a variety of such events. A detailed list of the differentially deleted genes, corresponding to Fig. 2, is provided in the Table S1.

**Figure 3:**
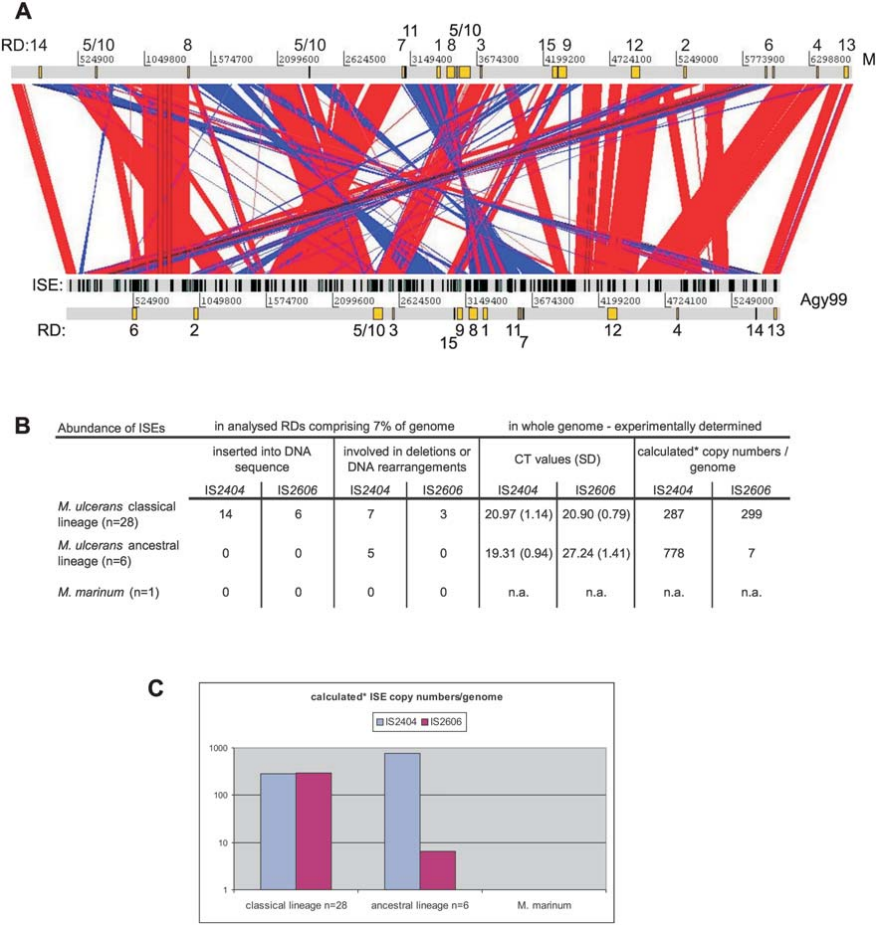
Involvement of ISEs in genomic diversity. A) Positions of the RDs and ISEs on the genomes of *M. marinum* M (top) and *M. ulcerans* Agy99 (bottom). RDs1-15 are located at different positions upon alignment of the two genomes, and some RDs occur on several loci on the *M. marinum* sequence due to genomic rearrangement and dislocations that formed the *M. ulcerans* Agy99 genome. The ISEs IS*2404* and IS*2606* are indicated as black bars in the marked lane. Sequence comparison was illustrated using the Artemis Comparison Tool software release 5 [31]. B) Test for whole genome abundance of IS*2404* and IS*2606* throughout a world-wide *M. ulcerans* strain collection (n = 34) and *M. marinum*. Indicated are the numbers of observed ISE involvements in the analysed 7% of the genome and the experimentally determined copy numbers. Whereas *M. marinum* M and water controls were devoid of ISEs, the abundance of IS*2404* and IS*2606* was measured between the lineages by quantitative real-time PCR. C) CT values were normalized using a unique gene target to account for differences in template input and calculated into copy numbers/genome. *These values reflect approximate numbers calculated from obtained CT values. Note that minor changes in CT value differences result in dramatic changes of determined copy numbers, i.e. the retrieved calculated values for the IS*2606* in the classical lineage, for which a genome information is available, deviate by a factor of three.

RDs 1 through 15 are evenly distributed on the genome as shown in Fig. 2. An overlay of positions of both ISEs and RDs (Fig. 3A) for the whole genome sequences of *M. marinum* M and *M. ulcerans* Agy99 shows that most RDs are associated with the presence of ISEs. Comparison of the two *M. ulcerans* lineages throughout RDs1 to 15 revealed a difference in ISE abundance (Fig. 2 and 3B), and Southern hybridization of representatives of the two lineages already indicated significant differences for IS*2606* [32]. We therefore compared the number of whole genome IS*2404* and IS*2606* copies by quantitative real-time PCR (Fig. 3B, C). The estimated mean difference between the classical and ancestral lineage for IS*2404* signals was 1.66 (95% CI = 0.64 to 2.68), indicating that the pronounced difference in abundance of IS*2404* between the two lineages was largely restricted to the analysed RDs. However, for IS*2606* an elevated CT value (27.24) was measured in the ancestral lineage resulting in an estimated mean difference between the lineages of 6.34 (95% CI = 4.87 to 7.81). This reflects a very low abundance of IS*2606* in the whole genome of strains of the ancestral lineage, explaining the observed lack of IS*2606* involvement in genome rearrangements in this lineage.

**Figure 4:**
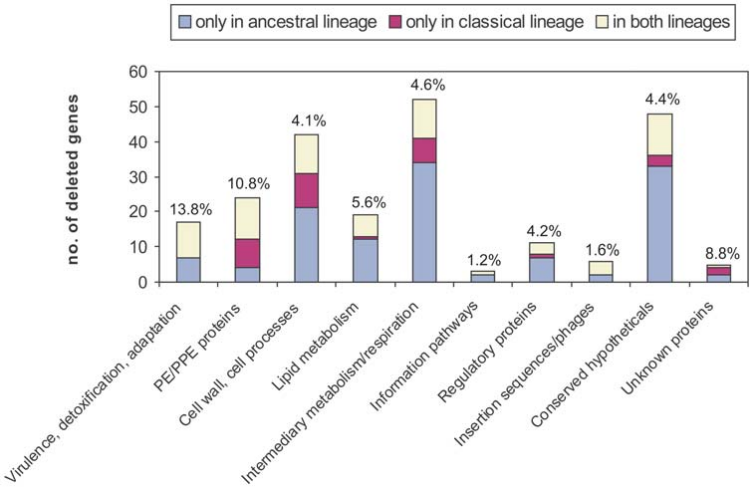
Numbers of CDSs deleted in *M. ulcerans* lineages. CDSs were subdivided in being deleted only in the ancestral lineage (light gray), only in the classical lineage (dark gray), or in both lineages (white). The percentage of silenced CDSs per functional category when set in relation to the respective number of genes in the whole genome is indicated above the bars.

The investigated RDs comprise in their ∼400 kbp DNA sequence 338 genes with respect to the *M. marinum* M sequence. Altogether 229 genes were found to be affected by differential inactivation. While a number of these genes was lost or inactivated only in one of the haplotypes (32 in the classical lineage), a large fraction (156) of the genes were silenced by independent events in two or more haplotypes (Fig. 4; for a comprehensive list see Fig. 2 and Table S1). This gene repertoire constitutes a broad spectrum of genomic variation on CDS level in the otherwise genetically monomorphic *M. ulcerans*. Subdivision of the lost or pseudogenized CDSs into functional protein categories (Fig. 4) showed that i) proteins lost only in the ancestral lineage belong predominantly to the functional categories cell wall/cell processes, lipid metabolism, intermediary metabolism/respiration and regulatory proteins; and ii) for the proteins lost in both lineages the categories virulence/detoxification/adaptation and PE/PPE proteins are overrepresented. When set in relation to the number of genes allocated to the functional categories in the whole genome, over 10% of all virulence/detoxification/adaptation and PE/PPE protein genes have been inactivated in one or both lineages alone in the analysed 7% of the genome (Fig. 4). We identified four regions of preferential genome instability (RDs9, 12, 13, 14) with twelve CDSs that were inactivated by three different events in the haplotypes analysed (Table 1). Seven of these CDSs are coding for proteins likely to interact with the environment/host of the bacterial cells (secreted or membrane proteins and PE/PPE proteins). Three of the CDSs are involved in the mycobacterial ESX-1 secretion apparatus, and embR_1 which is involved in cell wall biosynthesis.

**Table 1:** CDSs inactivated in at least three independent events in *M. ulcerans* haplotypes. Indicated are the *M. marinum* and *M. ulcerans* annotations.

RD	Coding sequence	Functional classification	Description
9	MMAR_3540	cell wall / cell processes	conserved hypothetical secreted protein
9	MMAR_3541		conserved membrane protein
9	MMAR_3542, MUL_2773		conserved hypothetical membrane protein with mechanosensitive domain identity
9	embR_1	regulatory proteins	transcriptional regulatory protein, probably regulating biosynthesis of the mycobacterial cell wall arabinan and resistance to ethambutol (Emb), regulating EmbA and EmbB
9	PPE5	PE/PPE	PPE family protein
12	MMAR_3884, MUL_3845		PE/PPE family protein
12	PPE2		PE/PPE family protein
12	MMAR_3990, MUL_3851		PE-PGRS protein
12	MMAR_3993, MUL_3855	intermediary metabolism / respiration	acyl-CoA dehydrogenase
13	esxB	virulence / detoxification / adaptation	10 kDa culture filtrate antigen cfp10, Esat-6 like protein, component of a novel secretion apparatus
14	esxB_1		culture filtrate antigen that forms part of a novel secretion apparatus
14	esxA_1		6 kDa culture filtrate antigen Esat6

## Discussion

Deletions are unidirectional events that serve as irreversible genetic and evolutionary markers, and their characterisation has repeatedly proven to be a powerful tool for phylogenetic analysis of mycobacteria and studies of their global and regional epidemiology [2],[7],[33]. The described polymorphisms in RDs1 to 15 can be used to distinguish *M. ulcerans* haplotypes and to position newly identified isolates in the established evolutionary scenario [9]. In the composition of their RDs, *M. ulcerans* members of the ancestral lineage resemble much more strain *M. marinum* M than *M. ulcerans* strains of the classical lineage [9]. Therefore, alignments of their genomic sequence to the *M. marinum* M sequence provided a clearer picture of the phylogeny than a mere comparison of the sequences of the *M. ulcerans* lineages. The detailed analysis of the RDs provides a repertoire of genes differentially silenced between the *M. ulcerans* haplotypes from different geographic origins.

The observed loss of genes supports findings that *M. ulcerans* lineages are undergoing reductive evolution to become niche-adapted specialists [8],[11]. Loss of gene functions under conditions of habitat changes may just be tolerated due to decreased requirement as compared to a generalist ancestor. However, in contrast to such random loss, several observations in this present analysis of 400 kbp of the *M. ulcerans* genome infer a selective advantage of loss of expression of particular genes: i) the identification of hot spot regions of genome instability, ii) the clustering of silenced genes into functional categories, and iii) the inactivation of a bulk of genes in different haplotypes by independent events that exceeds what is expected by chance alone. Some of these doubly or haplotype specific deleted CDSs might turn out to be patho-adaptive or anti-virulence genes although experimental work has to verify this hypothesis. There is compelling evidence for this to be a real phenomenon from studies in other mycobacteria [29]. For example, mutations at different positions of echA13 (also found in this study) and two other genes among a selection of mycolactone producing mycobacteria already led to the assumption of an independent, purifying selection [32]. Some of the identified gene products in RDs1-15 are likely to influence interaction of mycobacteria with the environment (e.g. members of the PE/PPE protein family and dehydrogenases, in part determining the cell wall lipid composition) or are known antigens in *M. tuberculosis* (e.g. the esx family proteins, Mpt63, and HspX). As already suspected for *M. africanum* and *M. ulcerans* [13],[34], the expression of esxA/esxB and/or HspX may be detrimental in a changing habitat or upon exposure to immune pressure. In hypervirulent strains of *M. tuberculosis* deletions in metabolic enzymes, cell surface-exposed proteins or regulators that respond to environmental stimuli have been identified [26]–[28]. For example, disruption of the *mce1* operon or regulators thereof possibly modulates the host's proinflammatory response and accelerates an immunopathological response in mice [28],[35]. Also, an *M. tuberculosis* orthologue of embR_1, in this study identified as being three times independently disrupted, closely interacts with PknH whose deletion was shown to result in a hypervirulent phenotype [26],[36]. Thus, genes listed in Table 2 should be among the first to be investigated for their role in patho-adaptation of *M. ulcerans*. Interestingly, no orthologues of the differentially silenced CDSs with known function listed in Table 2 are found in the genome sequence of *M. leprae* TN. After the description of the genome sequence of the African isolate Agy99 [11], this list of candidate anti-virulence genes constitutes a further step towards the description of the virulome of *M. ulcerans*.

**Table 2:** CDSs inactivated by independent mechanisms in either both lineages or only in the classical lineage depict candidates for virulence and adaptation.

Functional group (total no. of candidate CDS)	*M. marinum*/*M. ulcerans* coding sequence	Known or supposed function	in organism	Reference
Virulence, detoxification, adaptation (10)	ahpC_1	alkyl hydroperoxide reductase C protein, involved in oxidative stress response, associated with virulence and isoniazid resistance	*M. tuberculosis*	[41],[42]
	esxA, esxA_1, esxA_3, esxB, esxB_1	culture filtrate antigens 6 kDa early secretory antigenic target Esat-6/ 10 kDa culture filtrate antigen cfp10, components of a novel secretion apparatus, EsxA and EsxB form a heterodimer, involved in virulence or immune evasion	*M. tuberculosis*	[13],[43],[44]
			*M. marinum*	
			*M. ulcerans*	
	HspX_1/acr	α-crystallizable protein, heat shock protein, molecular chaperone, immunogenic protein but might impair microbe persistence	*M. tuberculosis*	[13],[45],[46]
			*M. ulcerans*	
	mce3A, mce3C, yrbE3B	Mce-family proteins, probably involved in host cell invasion and granuloma formation/ YrbE family protein, probably part of mce3 operon; mce operons affect virulence in M. t.	*M. tuberculosis*	[28],[47]
PE/PPE proteins (20)	MMAR_0186, MMAR_2894*, MMAR_2895*, MMAR_5447*, MMAR_5448*, MUL_0965, MUL_2203, MUL_3845, PPE2, PPE5, PPE51_2	PE/PPE family proteins, probably surface exposed and antigenic, source of genetic diversity	mycobacteria	[48],[49]
	MMAR_1161*, MMAR_1162*, MMAR_2969, MMAR_2970, MMAR_2973, MUL_0537*, MUL_0973/75/78*, MUL_2207, MUL_3851	PE-PGRS family proteins, probably surface exposed and antigenic		
Cell wall, cell processes (21)	MMAR_2839*/Mpt63	16 kDa immunogenic extracellular protein	*M. tuberculosis*	[50]
	MMAR_2841, MMAR_2843, MMAR_2898*, MMAR_3506* MMAR_3541, MUL_2187*, MUL_2212*, MUL_2222, MUL_2226, MUL_2773, MUL_2785, MUL_5047*	conserved hypothetical membrane or transmembrane proteins, probably surface exposed		
	MMAR_2842 MUL_2900	ion dependent/ short-chain fatty acid transporter		
	MMAR_2890*, MMAR_2891*, MMAR_2897*, MMAR_3540	conserved hypothetical secreted proteins, potentially antigenic		
	MMAR_2896*	WcaG-like nucleoside-diphosphate-sugar epimerase, NAD dependend dehydratase, possibly involved in cell wall biogenesis; epimerases may be potential drug targets in M. t.; WcaG is a synthesis gene for the capsule component fucose, virulence factor in K. p.	*M. tuberculosis, Klebsiella pneumoniae*	[51],[52]
	MUL_3838	drug-transport integral membrane protein, involved in drug resistance by an export mechanism		
Lipid metabolism (8)	MUL_2981	(Nrp, Non-ribosomal) peptide synthetase, involved in lipid metabolism		
	echA13, MUL_3841, MUL_3875, fadD9, fadE34_1*, MUL_3877	enoyl-CoA hydratase EchA13, acyl-CoA dehydrogenases, and fatty-acid-CoA ligases, involved in lipid degradation, probably all part of the catabolic repertoire; fadE gene deletions attenuate intracellular stage in M.t.	*M. tuberculosis*	[53],[54]
	MUL_2908, MUL_3865, MMAR_3529*	dehydrogenase/reductase/decarboxylase proteins		
Intermediary metabolism/respiration (18)	aldA_2*	NAD dependent aldehyde dehydrogenase oxidizes a variety of aldehydes		
	MUL_3855	acyl-CoA dehydrogenase		
	MUL_2221	fad flavoprotein Gmc dehydrogenase /oxidoreductase		
	MMAR_3505*	hydrolase; proteases are potential virulence factors		
	MMAR_2859/MUL_2895	(Fe-S) oxidoreductase		
	atsD_2	arylsulfatase, important for mineralization of sulfates		
	MUL_2204	1-aminocyclopropane-1-carboxylate deaminase		
	cyp138A4P, cyp278A1P	cytochrome P450 system proteins, heme-thiolate monooxygenase; the cytochrome P450 system is involved in lipid biosynthesis, hydroxylation of metabolites and detoxification of xenobiotics; a cytochrome P450 homolog gene deletion attenuates intracellular stage in M.t.	*M. tuberculosis*	[53],[54]
	glnA3*	glutamine synthetase, involved in glutamine biosynthesis, nitrogen metabolism		
	MUL_2211*	carbohydrate kinase		
	MUL_2213*	muconolactone isomerase, possibly involved in catechol catabolism in the beta-ketoadipate pathway		
	MUL_0963*	glycosyl transferase, probably involved in cellular mechanism, glycogen synthase		
	MUL_2224	O-methyltransferase		
	MUL_2764	amidotransferase		
Information pathways (1)	MUL_2206	HrpA-like helicase		
Regulatory protein (4)	embR_1	transcriptional regulatory protein, probably regulating biosynthesis of the mycobacterial cell wall arabinan and resistance to ethambutol, regulating EmbA and EmbB, associated with drug resistance in M.t.. EmbR in M.t. interacts with PknH whose deletion results in a hypervirulent phenotype	*M. tuberculosis*	[26],[36]
	hspR_2*	heat shock protein transcriptional repressor/regulator, homolog of the Acr heat shock regulon	*M. tuberculosis*	[46],[55]
	MUL_2779, MMAR_2939	possibly involved in transcription		
insertion sequences (4)	MUL_3219, MUL_3222, MUL_3223, MUL_3225	ype I restriction-modification system restriction subunits/ DNA methylases		
Conserved hypotheticals (15)	MMAR_2892*, MMAR_2952*, MUL_2193, MUL_2199, MUL_2201, MUL_2218, MUL_2302*, MUL_2311, MUL_2884, MUL_2897, MUL_2903, MUL_3228, MUL_3880, MUL_5038, MUL_5041	conserved hypothetical proteins		
Unknown proteins (3)	MMAR_0190, MMAR_2778*, MMAR_2893*	hypothetical proteins		

*: deleted only in the classical lineage. CDS are specified along the *M. ulcerans* Agy99 annotation, where possible, and the *M. marinum* M annotation where no *M. ulcerans* orthologues exist. An extended list including all 229 genes disrupted throughout RDs1-15 is supplied in Table S1.

It was earlier suspected that the ancestral and the classical lineage of *M. ulcerans* inherit different virulence potential [9],[16], and further evidence for that was provided by a recent study in Peru where Buruli ulcer cases are scarce despite frequent contact of people to *M. ulcerans* contaminated water bodies [15]. It is conceivable that the pronounced genome contraction that is specific to the classical lineage reflects a particular adaptation of this lineage. In particular, silencing of ten (of 21) CDSs of the functional category cell wall/cell processes and seven (of 18) of the group intermediary metabolism/respiration was confined to the classical lineage (Table 2). They fall either in the category of putative candidates for immune evasion upon loss (e.g. members of the esx gene family, Mpt63, the WcaG-like epimerase MMar_2896) or are of potential regulatory relevance, like hspR_2, a probable heat shock transcriptional repressor. CDSs for PE/PPE proteins were predominantly silenced in the classical lineage within the examined 7% of the *M. ulcerans* genome. However, when we investigated the strictly ISE-mediated disruptions and deletions *in silico* in the entire genome we found that ISEs pseudogenized 25% of all PE/PPE genes in strain Agy99 (not shown). The fact that members of this protein family were highly affected by genome shrinkage [11] suggests a particular importance for reducing expression of such surface exposed proteins.

**Figure 5:**
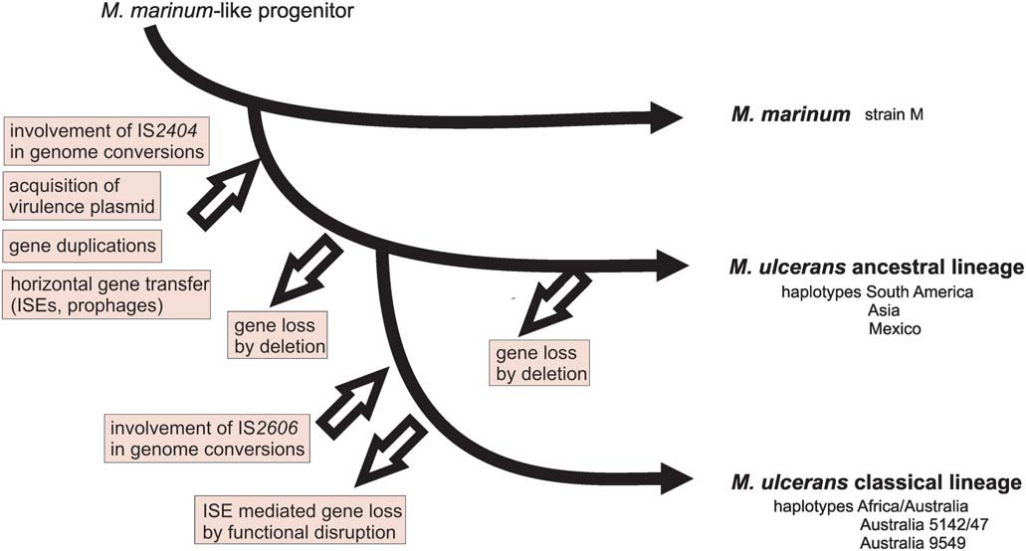
Genomic events leading to strain variations and pathogen emergence in *M. ulcerans* haplotypes.

*M. marinum* causes only occasionally ulcerative but self-healing infections in humans [37]. Without doubt, the acquisition of the virulence plasmid and the expression of the macrolide toxin mycolactone was an important step in the development of the ancestor of *M. ulcerans* to a mammalian pathogen [17]. On the other hand, other mycolactone producing mycobacteria closely related to *M. marinum* and *M. ulcerans* have been recently isolated from lesions in frogs and fish [38],[39] but so far not from infected humans. This indicates that additional factors contribute to the high virulence of the classical lineage of *M. ulcerans*. Our data indicate that, in addition to “gain of function” by acquisition of the virulence plasmid, loss of distinct anti-virulence genes, partly driven by ISE – in particular IS*2606* – expansion, might have equipped the classical lineage with a particular virulence and transmissibility (Fig. 5). It would be interesting to experimentally verify this hypothesis by testing these newly identified anti-virulence candidates in an appropriate *in vivo* model.

## Supporting Information

Table S1 CDSs inactivated in RDs1 through 15 across the *M. ulcerans* haplotypes. CDSs are listed in the order of the *M. marinum* annotation. Note that only CDSs are listed where *M. ulcerans* haplotypes differ from each other. Thus, not all MURDs distinguishing the classical lineage from *M. marinum* in these regions are mentioned but are found elsewhere [11]. All CDSs deleted in more than one haplotype were lost in independent events except when indicated (* = probably not independently deleted). When deleted or pseudogenized, CDSs are indicated in the *M. ulcerans* Agy99 annotation, where possible, and in the *M. marinum* M annotation where no *M. ulcerans* orthologue exists. When found present, respective CDSs are indicated as “present”. CDSs where no *M. marinum* orthologue exists are indicated “na” ( = not applicable). The Mexican haplotype could not be tested for all RDs that affected other haplotypes, as indicated “nd”( = not determined), therefore, the number of CDSs deleted in the Mexican haplotype is underestimated.
(0.04 MB PDF)

## References

[1] Alland D, Lacher DW, Hazbon MH, Motiwala AS, Qi W (2007). Role of large sequence polymorphisms (LSPs) in generating genomic diversity among clinical isolates of *Mycobacterium tuberculosis* and the utility of LSPs in phylogenetic analysis.. J Clin Microbiol.

[2] Brosch R, Gordon SV, Marmiesse M, Brodin P, Buchrieser C (2002). A new evolutionary scenario for the *Mycobacterium tuberculosis* complex.. Proc Natl Acad Sci U S A.

[3] Cole ST (2002). Comparative and functional genomics of the *Mycobacterium tuberculosis* complex.. Microbiology.

[4] Filliol I, Motiwala AS, Cavatore M, Qi W, Hazbon MH (2006). Global phylogeny of *Mycobacterium tuberculosis* based on single nucleotide polymorphism (SNP) analysis: insights into tuberculosis evolution, phylogenetic accuracy of other DNA fingerprinting systems, and recommendations for a minimal standard SNP set.. J Bacteriol.

[5] Behr MA, Wilson MA, Gill WP, Salamon H, Schoolnik GK (1999). Comparative genomics of BCG vaccines by whole-genome DNA microarray.. Science.

[6] Gagneux S, Small PM (2007). Global phylogeography of *Mycobacterium tuberculosis* and implications for tuberculosis product development.. Lancet Infect Dis.

[7] Hirsh AE, Tsolaki AG, DeRiemer K, Feldman MW, Small PM (2004). Stable association between strains of *Mycobacterium tuberculosis* and their human host populations.. Proc Natl Acad Sci U S A.

[8] Rondini S, Käser M, Stinear T, Tessier M, Mangold C (2007). Ongoing genome reduction in *Mycobacterium ulcerans*.. Emerg Infect Dis.

[9] Kaser M, Rondini S, Naegeli M, Stinear T, Portaels F (2007). Evolution of two distinct phylogenetic lineages of the emerging human pathogen *Mycobacterium ulcerans*.. BMC Evol Biol.

[10] Stinear TP, Jenkin GA, Johnson PD, Davies JK (2000). Comparative genetic analysis of *Mycobacterium ulcerans* and *Mycobacterium marinum* reveals evidence of recent divergence.. J Bacteriol.

[11] Stinear TP, Seemann T, Pidot S, Frigui W, Reysset G (2007). Reductive evolution and niche adaptation inferred from the genome of *Mycobacterium ulcerans*, the causative agent of Buruli ulcer.. Genome Research.

[12] Stinear TP, Seemann T, Harrison PF, Jenkin GA, Davies JK (2008). Insights from the complete genome sequence of *Mycobacterium marinum* on the evolution of *Mycobacterium tuberculosis*.. Genome Research.

[13] Huber CA, Ruf MT, Pluschke G, Kaser M (2008). Independent loss of immunogenic proteins in *Mycobacterium ulcerans* suggests immune evasion.. Clin Vaccine Immunol.

[14] WHO (2008). Buruli ulcer: progress report, 2004–2008, Weekly epidemiological record..

[15] Guerra H, Palomino JC, Falconi E, Bravo F, Donaires N (2008). *Mycobacterium ulcerans* Disease, Peru.. Emerg Infect Dis.

[16] Mve-Obiang A, Lee RE, Portaels F, Small PL (2003). Heterogeneity of mycolactones produced by clinical isolates of *Mycobacterium ulcerans*: implications for virulence.. Infect Immun.

[17] Stinear TP, Mve-Obiang A, Small PL, Frigui W, Pryor MJ (2004). Giant plasmid-encoded polyketide synthases produce the macrolide toxin of *Mycobacterium ulcerans*.. Proc Natl Acad Sci U S A.

[18] Stinear TP, Pryor MJ, Porter JL, Cole ST (2005). Functional analysis and annotation of the virulence plasmid pMUM001 from *Mycobacterium ulcerans*.. Microbiology.

[19] Maurelli AT (2007). Black holes, antivirulence genes, and gene inactivation in the evolution of bacterial pathogens.. FEMS Microbiol Lett.

[20] Sokurenko EV, Hasty DL, Dykhuizen DE (1999). Pathoadaptive mutations: gene loss and variation in bacterial pathogens.. Trends Microbiol.

[21] Chain PS, Carniel E, Larimer FW, Lamerdin J, Stoutland PO (2004). Insights into the evolution of *Yersinia pestis* through whole-genome comparison with *Yersinia pseudotuberculosis*.. Proc Natl Acad Sci U S A.

[22] Maurelli AT, Fernandez RE, Bloch CA, Rode CK, Fasano A (1998). “Black holes” and bacterial pathogenicity: a large genomic deletion that enhances the virulence of *Shigella spp.* and enteroinvasive *Escherichia coli*.. Proc Natl Acad Sci U S A.

[23] Torres AG, Vazquez-Juarez RC, Tutt CB, Garcia-Gallegos JG (2005). Pathoadaptive mutation that mediates adherence of shiga toxin-producing *Escherichia coli* O111.. Infect Immun.

[24] Moore RA, Reckseidler-Zenteno S, Kim H, Nierman W, Yu Y (2004). Contribution of gene loss to the pathogenic evolution of *Burkholderia pseudomallei* and *Burkholderia mallei*.. Infect Immun.

[25] Prunier AL, Schuch R, Fernandez RE, Mumy KL, Kohler H (2007). nadA and nadB of *Shigella flexneri* 5a are antivirulence loci responsible for the synthesis of quinolinate, a small molecule inhibitor of *Shigella* pathogenicity.. Microbiology.

[26] Papavinasasundaram KG, Chan B, Chung JH, Colston MJ, Davis EO (2005). Deletion of the *Mycobacterium tuberculosis* pknH gene confers a higher bacillary load during the chronic phase of infection in BALB/c mice.. J Bacteriol.

[27] Parish T, Smith DA, Kendall S, Casali N, Bancroft GJ (2003). Deletion of two-component regulatory systems increases the virulence of *Mycobacterium tuberculosis*.. Infect Immun.

[28] Shimono N, Morici L, Casali N, Cantrell S, Sidders B (2003). Hypervirulent mutant of *Mycobacterium tuberculosis* resulting from disruption of the mce1 operon.. Proc Natl Acad Sci U S A.

[29] Ten Bokum AM, Movahedzadeh F, Frita R, Bancroft GJ, Stoker NG (2008). The case for hypervirulence through gene deletion in *Mycobacterium tuberculosis*.. Trends Microbiol.

[30] Rutherford K, Parkhill J, Crook J, Horsnell T, Rice P (2000). Artemis: sequence visualization and annotation.. Bioinformatics.

[31] Carver TJ, Rutherford KM, Berriman M, Rajandream MA, Barrell BG (2005). ACT: the Artemis Comparison Tool.. Bioinformatics.

[32] Yip MJ, Porter JL, Fyfe JA, Lavender CJ, Portaels F (3007). Evolution of *Mycobacterium ulcerans* and other mycolactone-producing mycobacteria from a common *Mycobacterium marinum* progenitor.. J Bacteriol.

[33] Smith NH, Gordon SV, Rua-Domenech R, Clifton-Hadley RS, Hewinson RG (2006). Bottlenecks and broomsticks: the molecular evolution of *Mycobacterium bovis*.. Nat Rev Microbiol.

[34] de Jong BC, Hill PC, Brookes RH, Gagneux S, Jeffries DJ (2006). *Mycobacterium africanum* elicits an attenuated T cell response to early secreted antigenic target, 6 kDa, in patients with tuberculosis and their household contacts.. J Infect Dis.

[35] Uchida Y, Casali N, White A, Morici L, Kendall LV (2007). Accelerated immunopathological response of mice infected with *Mycobacterium tuberculosis* disrupted in the mce1 operon negative transcriptional regulator.. Cell Microbiol.

[36] Sharma K, Gupta M, Krupa A, Srinivasan N, Singh Y (2006). EmbR, a regulatory protein with ATPase activity, is a substrate of multiple serine/threonine kinases and phosphatase in *Mycobacterium tuberculosis*.. FEBS J.

[37] Petrini B (2006). *Mycobacterium marinum*: ubiquitous agent of waterborne granulomatous skin infections.. Eur J Clin Microbiol Infect Dis.

[38] Ranger BS, Mahrous EA, Mosi L, Adusumilli S, Lee RE (2006). Globally distributed mycobacterial fish pathogens produce a novel plasmid-encoded toxic macrolide, mycolactone f.. Infect Immun.

[39] Ucko M, Colorni A, Kvitt H, Diamant A, Zlotkin A (2002). Strain variation in *Mycobacterium marinum* fish isolates.. Appl Environ Microbiol.

[40] The Wellcome Trust Sanger Institute *Mycobacterium marinum* sequencing project [http://www.sanger.ac.uk/Projects/M_marinum/]

[41] Jaeger T (2007). Peroxiredoxin systems in mycobacteria.. Subcell Biochem.

[42] Nusrath UA, Selvakumar N, Narayanan S, Narayanan PR (2008). Molecular analysis of isoniazid-resistant clinical isolates of *Mycobacterium tuberculosis* from India.. Int J Antimicrob Agents.

[43] Brodin P, Rosenkrands I, Andersen P, Cole ST, Brosch R (2004). ESAT-6 proteins: protective antigens and virulence factors?. Trends Microbiol.

[44] Collins DM, Skou B, White S, Bassett S, Collins L (2005). Generation of attenuated *Mycobacterium bovis* strains by signature-tagged mutagenesis for discovery of novel vaccine candidates.. Infect Immun.

[45] Roupie V, Romano M, Zhang L, Korf H, Lin MY (2007). Immunogenicity of eight dormancy regulon-encoded proteins of *Mycobacterium tuberculosis* in DNA-vaccinated and tuberculosis-infected mice.. Infect Immun.

[46] Stewart GR, Newton SM, Wilkinson KA, Humphreys IR, Murphy HN (2005). The stress-responsive chaperone alpha-crystallin 2 is required for pathogenesis of *Mycobacterium tuberculosis*.. Mol Microbiol.

[47] Gioffre A, Infante E, Aguilar D, Santangelo MP, Klepp L (2005). Mutation in mce operons attenuates *Mycobacterium tuberculosis* virulence.. Microbes Infect.

[48] Brosch R, Pym AS, Gordon SV, Cole ST (2001). The evolution of mycobacterial pathogenicity: clues from comparative genomics.. Trends Microbiol.

[49] Fleischmann RD, Alland D, Eisen JA, Carpenter L, White O (2002). Whole-genome comparison of *Mycobacterium tuberculosis* clinical and laboratory strains.. J Bacteriol.

[50] Lyashchenko K, Colangeli R, Houde M, Al Jahdali H, Menzies D (1998). Heterogeneous antibody responses in tuberculosis.. Infect Immun.

[51] Kantardjieff KA, Kim CY, Naranjo C, Waldo GS, Lekin T (2004). *Mycobacterium tuberculosis* RmlC epimerase (Rv3465): a promising drug-target structure in the rhamnose pathway.. Acta Crystallogr D Biol Crystallogr.

[52] Wu JH, Wu AM, Tsai CG, Chang XY, Tsai SF (2008). Contribution of fucose-containing capsules in *Klebsiella pneumoniae* to bacterial virulence in mice.. Exp Biol Med (Maywood).

[53] Chang JC, Harik NS, Liao RP, Sherman DR (2007). Identification of mycobacterial genes that alter growth and pathology in macrophages and in mice.. J Infect Dis.

[54] Cole ST, Brosch R, Parkhill J, Garnier T, Churcher C (1998). Deciphering the biology of *Mycobacterium tuberculosis* from the complete genome sequence.. Nature.

[55] Stewart GR, Wernisch L, Stabler R, Mangan JA, Hinds J (2002). Dissection of the heat-shock response in *Mycobacterium tuberculosis* using mutants and microarrays.. Microbiology.

