# Supplementary material for: Differential Gene Repertoire in Mycobacterium ulcerans Identifies Candidate Genes for Patho-Adaptation
Source: PLoS Negl Trop Dis. 2008 Dec 23;2(12):e353. doi: 10.1371/journal.pntd.0000353 (PMC2600814; doi:10.1371/journal.pntd.0000353)
Supplement: Table S1 — CDSs inactivated in RDs1 through 15 across the M. ulcerans haplotypes. CDSs are listed in the order of the M. marinum annotation. Note that only CDSs are listed where M. ulcerans haplotypes differ from each other. Thus, not all MURDs distinguishing the classical lineage from M. marinum in these regions are mentioned but are found elsewhere [11]. All CDSs deleted in more than one haplotype were lost in independent events except when indicated (* = probably not independently deleted). When deleted or pseudogenized, CDSs are indicated in the M. ulcerans Agy99 annotation, where possible, and in the M. marinum M annotation where no M. ulcerans orthologue exists. When found present, respective CDSs are indicated as “present”. CDSs where no M. marinum orthologue exists are indicated “na” ( = not applicable). The Mexican haplotype could not be tested for all RDs that affected other haplotypes, as indicated “nd”( = not determined), therefore, the number of CDSs deleted in the Mexican haplotype is underestimated. (0.04 MB PDF) [file pntd.0000353.s001.pdf]

RD	MURD	*M. marinum* ortholog	Deleted in classical lineage	Deleted in ancestral lineage, haplotype			Functional classification	Description
				South America	Asia	Mexico		
14	4	MMAR_0183	present	MMAR_0183	present	nd	PE/PPE	PE-PGRS family protein
14	4	MMAR_0184	present	MMAR_0184	present	nd	cell wall / cell processes	conserved hypothetical membrane protein
14	4	PE35	present	PE35	present	nd	PE/PPE	PE family protein
14	4	MMAR_0186	MMAR_0186	MMAR_0186	nd	nd	PE/PPE	PPE family protein
14	4	esxB_1	esxB_1	esxB_1	esxB_1	nd	virulence/detox./adaptation	culture filtrate antigen that forms part of a novel secretion apparatus
14	4	esxA_1	esxA_1	esxA_1	esxA_1	nd	virulence/detox./adaptation	6 kDa culture filtrate antigen Esat6
14	4	esxA_3	esxA_3	esxA_3	nd	nd	virulence/detox./adaptation	conserved hypothetical EsxA-like protein
14	4	MMAR_0190	MMAR_0190	MMAR_0190	nd	nd	hypothetical protein	unknown
14	4	PPE51_2	PPE51_2	PPE51_2	present	nd	PE/PPE	PPE family protein
5,10	7	MMAR_0570	MUL_2211	present	present	present	interm. metabolism/respir.	C-term carbohydrate kinase
5,10		MMAR_0569	MUL_2212	present	present	present	cell wall / cell processes	conserved hypothetical transmembrane protein
5,10		MMAR_0568	MUL_2213	present	present	present	interm. metabolism/respir.	muconolactone isomerase, possibly involved in catechol catabolism in the beta-ketoadipate pathway
8	25	MMAR_1161	MMAR_1161	nd	present	nd	PE/PPE	PE-PGRS family protein
8	25	MMAR_1162	MMAR_1162	nd	present	nd	PE/PPE	PE-PGRS family protein
5,10		fadE34_1	fadE34_1	present	present	present	lipid metabolism	function unknown, but involved in lipid degradation
7	58	na	present	MUL_3218	present	present	insertion seqs and phages	zinc metalloprotease
7		na	MUL_3219	MUL_3219	present	present	insertion seqs and phages	ype I restriction-modification system restriction subunit
7		na	MUL_3222	MUL_3222	present	present	insertion seqs and phages	C-term restriction endonuclease S subunit
7		na	MUL_3223	MUL_3223	present	present	insertion seqs and phages	ype I restriction/modification system DNA methylase
7		na	MUL_3225	MUL_3225	present	present	insertion seqs and phages	N-term fragment of type I restriction/modification system DNA methylase
7		na	MUL_3228	MUL_3228	present	present	conserved hypotheticals	C-term conserved hypothetical protein, present in full on the MU plasmid, pMUM001
11		MMAR_2557	present	present	MUL_3204	present	interm. metabolism / respir.	aldose 1-epimerase, unknown role in carbohydrate transport and metabolism
11		MMAR_2558	present	present	MUL_3203	MUL_3203*	conserved hypotheticals	conserved hypothetical protein
11		MMAR_2559	present	present	MUL_3202	MUL_3202*	interm. metabolism / respir.	conserved hypothetical oxidoreductase
11		MMAR_2560	present	present	MUL_3201	MUL_3201*	regulatory proteins	transcriptional regulatory protein, probably GntR-family
11		MMAR_2561	present	present	MUL_3200	MUL_3200*	conserved hypotheticals	conserved hypothetical protein
11		MMAR_2562	present	present	MUL_3199	MUL_3199*	cell wall / cell processes	conserved hypothetical membrane protein
11		MMAR_2563	present	present	MUL_3198	MUL_3198*	information pathways	translation initiation inhibitor, may be involved in inhibition of protein synthesis by cleavage of mRNA
1		glnA3	glnA3	present	present	nd	interm. metabolism / respir.	glutamine synthetase GlnA3
1	62	MMAR_2766	MUL_2981	MUL_2981	present	nd	lipid metabolism	(Nrp, Non-ribosomal) peptide synthetase, involved in lipid metabolism
1		MMAR_2767	present	MUL_2980	present	nd	conserved hypotheticals	conserved hypothetical protein
1		cyp140A5	present	cyp140A5	present	nd	interm. metabolism / respir.	cytochrome P450 Cyp140A5, heme-thiolate monooxygenase protein, probably involved in oxidization of a variety of structurally unrelated compounds including steroids, fatty acids, and xenobiotics
1		lppE	present	lppE	present	nd	cell wall / cell processes	conserved lipoprotein LppE
1		MMAR_2770	present	MUL_2977	present	nd	interm. metabolism / respir.	short-chain type dehydrogenase/reductase
1		MMAR_2771	present	MUL_2976	present	nd	conserved hypotheticals	conserved hypothetical protein
1		MMAR_2778	MMAR_2778	present	present	nd	unknown	hypothetical protein
8	72	MMAR_2839	MMAR_2839	present	present		cell wall / cell processes	16 kDa immunogenic extracellular protein Mpt63, unknown function
8	72	atsD_2	atsD_2	present	present	atsD_2	interm. metabolism / respir.	arylsulfatase AtsD_2, important for mineralization of sulfates
8	72	MMAR_2841	MMAR_2841	present	present	MMAR_2841	cell wall / cell processes	conserved hypothetical membrane protein
8	72	MMAR_2842	MMAR_2842	present	present	MMAR_2842	cell wall / cell processes	unknown, domain identity suggests ion dependent transporter
8	72	MMAR_2843	MMAR_2843	present	present	MMAR_2843	cell wall / cell processes	conserved hypothetical membrane protein

RD	MURD	*M. marinum* ortholog	Deleted in classical lineage	Deleted in ancestral lineage, haplotype			Functional classification	Description
				South America	Asia	Mexico		
8		MMAR_2844	present	present	present	MUL_2914	interm. metabolism / respir.	metal-dependent hydrolase
8		ahpC_1	present	present	present	ahpC_1	virulence/detox./adaptation	alkyl hydroperoxide reductase C protein AhpC, involved in oxidative stress response
8		MMAR_2846	present	present	present	MUL_2911	interm. metabolism / respir.	alpha-L-fucosidase, involved in carbohydrate transport and metabolism
8		MMAR_2847	present	present	present	MUL_2910	unknown	hypothetical protein
8		MMAR_2848	present	present	present	MUL_2909	conserved hypotheticals	conserved hypothetical protein
8		MMAR_2849	MUL_2908	present	present	MUL_2908	interm. metabolism / respir.	short-chain type dehydrogenase/reductase
8		MMAR_2850	present	present	present	MUL_2907	conserved hypotheticals	conserved hypothetical protein
8		MMAR_2851	present	present	present	MUL_2906	conserved hypotheticals	conserved hypothetical protein, has weak identity with hydrolase domains
8		MMAR_2852	present	present	present	MUL_2905	conserved hypotheticals	conserved hypothetical protein, contains dihydrofolate reductase domain
8		MMAR_2853	present	present	present	MUL_2904	cell wall / cell processes	conserved hypothetical membrane protein
8		MMAR_2854	MUL_2903	present	present	MUL_2903	conserved hypotheticals	conserved hypothetical protein
8		MMAR_2855	present	present	present	MUL_2902	regulatory proteins	transcriptional regulatory protein
8		MMAR_2857	MUL_2900	present	present	MUL_2900	cell wall / cell processes	short-chain fatty acid transporter
8		none	MUL_2897	present	present	MUL_2897	conserved hypotheticals	conserved hypothetical protein
8		MMAR_2859	MUL_2895	present	present	MUL_2895	interm. metabolism / respir.	conserved hypothetical oxidoreductase, domain identity with Fe-S oxidoreductases
8		tpx	present	present	present	tpx	interm. metabolism / respir.	thiol peroxidase Tpx, has antioxidant activity, probably removes peroxides
8		fadE18	present	present	present	fadE18	lipid metabolism	acyl-CoA dehydrogenase FadE18, probably involved in lipid degradation
8		fadE17	present	present	present	fadE17	lipid metabolism	acyl-CoA dehydrogenase FadE17, probably involved in lipid degradation
8		echA13	echA13	present	present	echA13	lipid metabolism	enoyl-CoA hydratase EchA13, probably involved in oxidization of fatty acids
8		MMAR_2864	present	present	present	MUL_2890	interm. metabolism / respir.	oxygenase
8		MMAR_2865	present	present	present	MUL_2889	interm. metabolism / respir.	oxygenase, probably involved in electron transfer
8		ephB	present	present	present	ephB	virulence/detox./adaptation	Epoxide hydrolase EphB, acts on epoxides (alkene oxides, oxiranes), involved in xenobiotic detoxification, determines steady-state levels of physiological mediators
8		MMAR_2867	present	present	present	MUL_2887	interm. metabolism / respir.	conserved hypothetical oxidoreductase
8		ribA1	present	present	present	ribA1	interm. metabolism / respir.	Riboflavin biosynthesis protein RibA1
8		MMAR_2869	present	present	present	MUL_2885	interm. metabolism / respir.	short-chain type dehydrogenase/reductase
8		MMAR_2870	MUL_2884	present	present	MUL_2884	conserved hypotheticals	conserved hypothetical membrane protein
8		MMAR_2871	present	present	present	MUL_2882	conserved hypotheticals	conserved hypothetical protein
8		MMAR_2872	present	present	present	MUL_2881	cell wall / cell processes	multidrug transport integral membrane protein Mmr, maybe required for the translocation of the substrate across the membrane
8		mce3R	present	present	present	mce3R	regulatory proteins	transcriptional repressor Mce3R, probably TetR-family, repressor of the mce3 operon, probably also has regulatory action on the mce2 operon
8		MMAR_2874	present	present	present	MUL_2878	conserved hypotheticals	conserved hypothetical protein
8		MMAR_2875	present	present	present	MUL_2877	interm. metabolism / respir.	aldehyde dehydrogenase, probably oxidizing aliphatic and aromatic aldehydes
8		MMAR_2876	present	present	present	MUL_2876	regulatory proteins	conserved hypothetical regulatory protein, has weak domain identity with two-component response regulators
8		cyp278A1P	cyp278A1P	present	present	cyp278A1P	interm. metabolism / respir.	cytochrome P450 278A1 Cyp278A1P, heme-thiolate monooxygenase
8		MMAR_2878	present	present	present	MUL_2874	conserved hypothetical	conserved hypothetical protein, has domain identity with ketosteroid isomerase-related proteins
8		MMAR_2879	present	present	present	MUL_2873	interm. metabolism / respir.	ferredoxin, Fe-S protein involved in electron tranfer
8		yrbE3A	present	present	present	yrbE3A	virulence/detox./adaptation	conserved hypothetical integral membrane protein YrbE3A

RD	MURD	*M. marinum* ortholog	Deleted in classical lineage	Deleted in ancestral lineage, haplotype			Functional classification	Description
				South America	Asia	Mexico		
8		yrbE3B	yrbE3B	present	present	yrbE3B	virulence/detox./adaptation	conserved hypothetical integral membrane protein, YrbE family, probably part of mce3 operon
8		mce3A	mce3A	present	present	mce3A	virulence/detox./adaptation	Mce-family protein Mce3A, thought to be involved in host cell invasion
8		mce3B	present	present	present	mce3B	virulence/detox./adaptation	Mce-family protein Mce3B, thought to be involved in host cell invasion
8		mce3C	mce3C	present	present	mce3C	virulence/detox./adaptation	Mce-family protein Mce3C, thought to be involved in host cell invasion
8		mce3D	present	present	present	mce3D	virulence/detox./adaptation	Mce-family protein Mce3D, thought to be involved in host cell invasion
8		lprM	present	present	present	lprM	virulence/detox./adaptation	Mce-family lipoprotein LprM, thought to be involved in host cell invasion
8		mce3F	present	present	present	mce3F	virulence/detox./adaptation	Mce-family protein Mce3F, thought to be involved in host cell invasion
8		MMAR_2888	present	present	present	MUL_2864	cell wall / cell processes	conserved Mce associated membrane protein
8		MMAR_2889	present	present	present	MUL_2863	cell wall / cell processes	conserved Mce associated membrane protein
8	68	MMAR_2890	MMAR_2890	present	present	present	cell wall / cell processes	conserved hypothetical secreted protein
8	68	MMAR_2891	MMAR_2891	present	present	present	cell wall / cell processes	conserved hypothetical secreted protein
8	68	MMAR_2892	MMAR_2892	present	present	present	conserved hypotheticals	conserved hypothetical protein
8	68	MMAR_2893	MMAR_2893	present	present	present	unknown	hypothetical protein
8	68	MMAR_2894	MMAR_2894	present	present	present	PE/PPE	PE family protein
8	68	MMAR_2895	MMAR_2895	present	present	present	PE/PPE	PE family protein
8	68	MMAR_2896	MMAR_2896	present	present	present	cell wall / cell processes	nucleoside-diphosphate-sugar epimerase WcaG-like, possibly involved in cell wall biogenesis
8	68	MMAR_2897	MMAR_2897	present	present	present	cell wall / cell processes	conserved hypothetical secreted protein
8	68	MMAR_2898	MMAR_2898	present	present	present	cell wall / cell processes	conserved hypothetical membrane protein
5,10		betP	present	present	present	betP	cell wall / cell processes	high-affinity uptake of glycine betaine, supposed responsible for the translocation of the substrate across the membrane
5,10	71	MMAR_2917	MUL_2187	present	present	present	cell wall / cell processes	conserved hypothetical membrane protein
5,10		MMAR_2935	MUL_2193	present	present	MUL_2193	conserved hypotheticals	conserved hypothetical protein
5,10		fadD9_1	fadD9	present	present	fadD9	lipid metabolism	fatty-acid-CoA ligase FadD9, involved in lipid degradation
5,10		MMAR_2937	present	present	present	MUL_2197	conserved hypotheticals	conserved protein
5,10		MMAR_2938	present	present	present	MUL_2198	interm. metabolism/respir.	nitroreductase
5,10	72	MMAR_2939	MMAR_2939	present	present	MMAR_2939	regulatory proteins	possibly involved in transcription
5,10		MMAR_2940	MUL_2199	present	present	MUL_2199	conserved hypotheticals	C-term conserved hypothetical protein
5,10		MMAR_2941	present	present	present	MUL_2200	regulatory proteins	membrane-anchored serine/threonine-protein kinase
5,10		MMAR_2942	MUL_2201	present	present	MUL_2201	conserved hypotheticals	conserved hypothetical protein
5,10		MMAR_2943	present	present	present	MUL_2202	conserved hypotheticals	conserved protein
5,10		MMAR_2944	MUL_2203	present	present	MUL_2203	PE/PPE	PPE family protein
5,10		MMAR_2945	MUL_2204	present	present	MUL_2204	interm. metabolism/respir.	1-aminocyclopropane-1-carboxylate deaminase
5,10		MMAR_2946	present	present	present	MUL_2205	conserved hypotheticals	conserved protein
5,10		MMAR_2947	MUL_2206	present	present	MUL_2206	information pathways	HrpA-like helicase
5,10		MMAR_2948	MUL_2207	present	present	MUL_2207	PE/PPE	N-term PE-PGRS family protein
5,10		MMAR_2949	present	present	present	MUL_2208	cell wall/cell processes	conserved hypothetical secreted protein
5,10		MMAR_2950	present	present	present	MUL_2209	interm. metabolism/respir.	oxidoreductase thought to be involved in fatty acid degradation. FadB and FadA are the alpha and BetA subunits of the multifunctional enzyme complex of the fatty acid degradation cycle
5,10		MMAR_2951	present	present	present	MUL_2210	cell wall / cell processes	chitinase/cellulase
5,10		MMAR_2952	MUL_2218	present	present	MUL_2218	conserved hypotheticals	conserved hypothetical protein
5,10		MMAR_2953	present	present	present	MUL_2220	cell wall / cell processes	conserved hypothetical secreted protein
5,10		MMAR_2954	MUL_2221	present	present	MUL_2221	interm. metabolism/respir.	C-term dehydrogenase fad flavoprotein Gmc, oxidoreductase
5,10	73	MMAR_2966	MUL_2222	present	present	MUL_2222	cell wall / cell processes	C-term conserved two-domain membrane protein

RD	MURD	*M. marinum* ortholog	Deleted in classical lineage	Deleted in ancestral lineage, haplotype			Functional classification	Description
				South America	Asia	Mexico		
5,10		MMAR_2967/68	present	present	present	MUL_2223	conserved hypotheticals	conserved hypothetical protein
5,10	74	MMAR_2969	MMAR_2969	present	present	MMAR_2969	PE/PPE	PE-PGRS family protein
5,10	74	MMAR_2970	MMAR_2970	present	present	MMAR_2970	PE/PPE	PE-PGRS family protein
5,10	74	MMAR_2971	MUL_2224	present	present	MUL_2224	interm. metabolism/respir.	O-methyltransferase
5,10	74	MMAR_2972	present	present	present	MMAR_2972	cell wall / cell processes	conserved hypothetical sugar transport protein
5,10	74	MMAR_2973	MMAR_2973	present	present	MMAR_2973	PE/PPE	PE-PGRS family protein
5,10	74	MMAR_2974	present	present	present	MMAR_2974	lipid metabolism	enoyl-CoA hydratase, possibly oxidizing fatty acids
5,10		MMAR_2975	MUL_2226	present	present	MUL_2226	cell wall / cell processes	conserved hypothetical membrane protein
5,10		hspR_2	hspR_2	present	present	present	regulatory proteins	C-term heat shock protein transcriptional repressor HspR_2
3		MMAR_3058	MUL_2302	present	present	present	conserved hypotheticals	conserved hypothetical protein
3		MMAR_3060	MMAR_3060	MMAR_3060	MMAR_3060	nd	cell wall / cell processes	conserved hypothetical membrane protein
3		MMAR_3061	(Au42/47)	MUL_2305	present	present	conserved hypotheticals	conserved hypothetical protein
3		MMAR_3062	(Au42/47)	MUL_2306	present	present	insertion seqs and phages	transposition of an insertion sequence
3		MMAR_3063	(Au42/47)	MUL_2307	present	present	cell wall / cell processes	conserved hypothetical membrane protein
3		MMAR_3064	(Au42/47)	MUL_2308	present	present	conserved hypotheticals	conserved membrane protein
3		MMAR_3065	(Au42/47)	MUL_2309	present	present	conserved hypotheticals	conserved hypothetical membrane protein
3		MMAR_3066	MUL_2311	MUL_2311	present	present	conserved hypotheticals	conserved hypothetical protein
15	92	HspX_1	HspX_1	present	present	HspX_1	virulence/detox./adaptation	heat shock protein, molecular chaperone
9	92	MMAR_3505	MMAR_3505	present	present	nd	interm. metabolism / respir.	conserved hypothetical hydrolase, involved in metabolism
9	92	MMAR_3506	MMAR_3506	present	present	nd	cell wall / cell processes	conserved hypothetical membrane protein
9	93	MMAR_3529	MMAR_3529	present	present	nd	interm. metabolism / respir.	dehydrogenase, involved in metabolism
9		MMAR_3530	present	present	MUL_2763	nd	conserved hypotheticals	conserved protein
9		MMAR_3531/32	MUL_2764	present	MUL_2764	nd	interm. metabolism / respir.	C-term amidotransferase family protein
9		MMAR_3533	present	present	MUL_2765	nd	interm. metabolism / respir.	methyltransferase (Methylase)
9		MMAR_3534	present	present	MUL_2766	nd	lipid metabolism	O-methyltransferase, possibly involved in polyketide biosynthesis
9		MMAR_3535	present	present	MUL_2767	nd	cell wall / cell processes	conserved hypothetical membrane protein
9		MMAR_3536	present	present	MUL_2768	nd	conserved hypotheticals	conserved hypothetical protein, high domain identity with epimerases and phenazine biosynthesis-like protein PhzC/PhzF
9		MMAR_3537	present	present	MUL_2769	nd	conserved hypotheticals	conserved hypothetical protein
9		MMAR_3538	present	present	MUL_2770	nd	cell wall / cell processes	conserved hypothetical membrane protein
9		MMAR_3538	present	present	MUL_2771	nd	cell wall / cell processes	conserved hypothetical membrane protein
9		MMAR_3539	present	MUL_2772	MUL_2772	nd	conserved hypotheticals	conserved hypothetical protein with ribonuclease I domain identity
9	94	MMAR_3540	MMAR_3540	MMAR_3540	MMAR_3540	nd	cell wall / cell processes	conserved hypothetical secreted protein
9	94	MMAR_3541	MMAR_3541	MMAR_3541	MMAR_3541	nd	cell wall / cell processes	conserved membrane protein
9	94	MMAR_3542	MUL_2773	MMAR_3542	MMAR_3542	nd	cell wall / cell processes	conserved hypothetical membrane protein with mechanosensitive domain identity
9		MMAR_3543	present	MUL_2774	MUL_2774	nd	cell wall / cell processes	conserved membrane protein; contains multiple functional domains including VWA domain (von Willebrand factor (vWF) type A domain found in extracellular proteins like integrins and mediates adhesion; contains a vault protein Inter-alpha-Trypsin domain
9		MMAR_3544	present	MUL_2775	MUL_2775	nd	conserved hypotheticals	conserved hypothetical protein
9		embR_1	embR_1	embR_1	embR_1	nd	regulatory proteins	transcriptional regulatory protein EmbR_1; probably regulating biosynthesis of the mycobacterial cell wall arabinan and resistance to ethambutol (Emb), regulating EmbA and EmbB
9	95	PPE5	PPE5	PPE5	PPE5	nd	PE/PPE	PPE family protein
9	95	MMAR_3547	MUL_2779	MUL_2779	nd	nd	regulatory proteins	regulatory protein

RD	MURD	*M. marinum* ortholog	Deleted in classical lineage	Deleted in ancestral lineage, haplotype			Functional classification	Description
				South America	Asia	Mexico		
9		MMAR_3548	present	MUL_2781	present	nd	conserved hypotheticals	conserved hypothetical protein
9		MMAR_3549	present	MUL_2782	present	nd	conserved hypotheticals	conserved hypothetical protein; contains glutamine synthetase catalytic domain
9		PE_PGRS39	present	PE_PGRS39	present	nd	PE/PPE	PE-PGRS family protein
9		MMAR_3551	present	MUL_2784	present	nd	cell wall / cell processes	conserved Proline, Glycine, Valine-rich secreted protein
9		MMAR_3552	MUL_2785	MUL_2785	present	nd	cell wall / cell processes	conserved hypothetical membrane protein
9		MMAR_3553	present	MUL_2786	present	nd	interm. metabolism / respir.	alanine-rich hydrolase; probably involved in cellular metabolism
9		MMAR_3554	present	MUL_2787	present	nd	cell wall / cell processes	conserved hypothetical membrane protein; possibly membrane transporter involved in transport of substrate (possibly cationic amino acids)
9		MMAR_3555	present	MUL_2788	present	nd	interm. metabolism / respir.	saccharopine dehydrogenase, domain identity with saccharopine dehydrogenase; in some organisms this enzyme is found as a bifunctional polypeptide with lysine ketoglutarate
9		MMAR_3556	present	MUL_2789	present	nd	interm. metabolism / respir.	aldehyde dehydrogenase NAD dependent; oxidizes catalytically a variety of aldehydes
9		MMAR_3557	present	MUL_2790	present	nd	regulatory proteins	transcriptional regulatory protein; probably Lrp/AsnC-family
9		MMAR_3558	present	MUL_2791	present	nd	interm. metabolism / respir.	aminotransferase (Adenosylmethionine-8-amino-7-oxononanoate), BioA domain identity, Coenzyme metabolism
9		MMAR_3559	present	MUL_2792	present	nd	conserved hypotheticals	conserved hypothetical protein, possible oxidoreductase
12		cyp138A4	cyp138A4P	cyp138A4P?	present	nd		cytochrome P450 138A4 Cyp138A4P
12	105	MMAR_3972	MMAR_3972	MMAR_3972	MMAR_3972	nd	interm. metabolism / respir.	non-ribosomal peptide synthetase
12		MMAR_3977	MUL_3838	MUL_3838	present	nd	cell wall / cell processes	drug-transport integral membrane protein, involved in drug resistance by an export mechanism
12		MMAR_3978	present	MUL_3839	present	nd	interm. metabolism / respir.	coenzyme F420-dependent oxidoreductase
12		MMAR_3979	present	MUL_3840	present	nd	lipid metabolism	acyl-CoA dehydrogenase, involved in lipid degradation
12		MMAR_3980	MUL_3841	MUL_3841	present	nd	lipid metabolism	acyl-CoA dehydrogenase, involved in lipid degradation
12		fadE17_2	present	fadE17_2	present	nd	lipid metabolism	acyl-CoA dehydrogenase FadE17_2, supposed lipid degradation
12		MMAR_3982	present	MUL_3843	present	nd	lipid metabolism	acyl-CoA dehydrogenase
12		MMAR_3983	present	MUL_3844	MUL_3844	nd	lipid metabolism	fatty-acid-CoA ligase
12		MMAR_3984	MUL_3845	MUL_3845	MUL_3845	nd	PE/PPE	PE/PPE family protein
12		MMAR_3985	present	MUL_3846	MUL_3846	nd	cell wall / cell processes	conserved hypothetical secreted protein
12		MMAR_3986	present	MUL_3847	MUL_3847	nd	conserved hypotheticals	conserved hypothetical membrane protein, contains a ricin-type beta-trefoil domain
12		MMAR_3987	present	MUL_3848	MUL_3848	nd	regulatory proteins	conserved hypothetical transcriptional regulatory protein
12		MMAR_3988	present	MUL_3849	MUL_3849	nd	interm. metabolism / respir.	conserved metal-dependent hydrolase
12		PPE2	PPE2	PPE2	PPE2	nd	PE/PPE	PE/PPE family protein
12		MMAR_3990	MUL_3851	MUL_3851	MUL_3851	nd	PE/PPE	PE-PGRS protein
12		rsbU	present	rsbU	rsbU	nd	information pathways	Regulator of sigma subunit, anti-anti-sigma factor RsbU
12		MMAR_3992	present	MUL_3854	MUL_3854	nd	lipid metabolism	acyl-CoA dehydrogenase
12		MMAR_3993	MUL_3855	MUL_3855	MUL_3855	nd	interm. metabolism / respir.	acyl-CoA dehydrogenase
12		MMAR_3994	present	MUL_3858	MUL_3858	nd	conserved hypotheticals	conserved hypothetical alanine-rich protein
12		MMAR_3995	present	MUL_3859	MUL_3859	nd	conserved hypotheticals	conserved hypothetical protein
12		cyp187A4	present	cyp273A3	cyp273A3	nd	interm. metabolism / respir.	cytochrome P450
12		adhB_1	present	adhB_1	adhB_1	nd	interm. metabolism / respir.	zinc-containing alcohol dehydrogenase NAD dependent AdhB_1, thought to catalyze the reversible oxidation of ethanol to acetaldehyde with the concomitant reduction of NAD. probably acts on primary or secondary alcohols or hemiacetals

RD	MURD	*M. marinum* ortholog	Deleted in classical lineage	Deleted in ancestral lineage, haplotype			Functional classification	Description
				South America	Asia	Mexico		
12		MMAR_3998	present	MUL_3862	MUL_3862	nd	interm. metabolism / respir.	short chain dehydrogenase
12		cyp108B4	present	present	cyp108F1	nd	interm. metabolism / respir.	cytochrome P450 108F1 Cyp108F1
12		accD4_2	present	present	accD4_2	nd	interm. metabolism / respir.	propionyl-CoA carboxylase beta chain 4, AccD4_2
12		MMAR_4001/02	MUL_3865	present	MUL_3865	nd	interm. metabolism / respir.	dehydrogenase/decarboxylase protein
12		MMAR_4003	present	present	MUL_3866	nd	interm. metabolism / respir.	oxidoreductase
12		MMAR_4004	present	present	MUL_3867	nd	conserved hypotheticals	conserved hypothetical protein, contains a Ntf2-like superfamily domain
12		MMAR_4005	present	present	MUL_3868	nd	cell wall / cell processes	conserved hypothetical secreted protein
12		MMAR_4006	present	present	MUL_3869	nd	conserved hypotheticals	conserved hypothetical protein, contains thioesterase/thiol ester dehydrase-isomerase superfamily domain
12		fadD19_2	present	present	fadD19_2	nd	lipid metabolism	fatty-acid-CoA ligase FadD19_2, involved in lipid degradation
12		cyp187A5	present	present	cyp273A4	nd	interm. metabolism / respir.	cytochrome P450 273A4 Cyp273A4
12		MMAR_4009	present	present	MUL_3873	nd	interm. metabolism / respir.	conserved hypothetical dehydratase
12		MMAR_4010	present	present	MUL_3874	nd	lipid metabolism	acyl-CoA dehydrogenase, involved in lipid degradation
12		MMAR_4011	MUL_3875	present	MUL_3875	nd	lipid metabolism	acyl-CoA dehydrogenase, involved in lipid degradation
12		MMAR_4012	present	present	MUL_3876	nd	interm. metabolism / respir.	conserved hypothetical metal-dependent hydrolase
12		MMAR_4013	MUL_3877	present	MUL_3877	nd	lipid metabolism	fatty-acid-CoA ligase, involved in lipid degradation
12		MMAR_4014	present	present	MUL_3878	nd	interm. metabolism / respir.	conserved hypothetical metal-dependent hydrolase
12		echA12_1	present	present	echA12_1	nd	lipid metabolism	enoyl-CoA hydratase, EchA12_1, oxidizes fatty acids using specific components (by similarity)
12		MMAR_4016	MUL_3880	present	MUL_3880	nd	conserved hypotheticals	conserved hypothetical protein
12		MMAR_4017	present	present	MUL_3882	nd	conserved hypotheticals	conserved hypothetical protein
12		MMAR_4018	present	present	MUL_3883	nd	unknown	hypothetical protein
2		MMAR_4226	MUL_0963	present	nd	nd	interm. metabolism / respir.	glycosyl transferase, probably involved in cellular mechanism
2	120	MMAR_4316	MUL_0973/75/78	present	nd	nd	PE/PPE	PE-PGRS family protein
2		MMAR_4319	present	MUL_0964	present	nd	PE/PPE	PPE family protein
2		MMAR_4320	MUL_0965	MUL_0965	present	nd	PE/PPE	PPE family protein
2		aldA_2	aldA_2	present	present	nd	interm. metabolism / respir.	aldehyde dehydrogenase NAD dependent AldA_2, oxidizes a variety of aldehydes
6		MMAR_4953	MUL_0537	present	present	present	PE/PPE	PE-PGRS family protein
4		acrA1_1	present	acrA1_1	present	nd	interm. metabolism / respir.	multi-functional enzyme with acyl-CoA-reductase activity AcrA1_1
4		MMAR_5273	present	MUL_4347	present	nd	conserved hypotheticals	conserved hypothetical membrane protein
13		MMAR_5445	MUL_5041	present	present	MUL_5041	conserved hypotheticals	conserved hypothetical protein
13	152	MMAR_5446	MUL_5038	present	present	MUL_5038	conserved hypotheticals	conserved hypothetical protein
13	152	MMAR_5447	MMAR_5447	present	present	MMAR_5447*	PE/PPE	PE family protein
13	152	MMAR_5448	MMAR_5448	present	present	MMAR_5448*	PE/PPE	PE family protein
13	152	esxB	esxB	present	esxB	esxB	virulence/detox./adaptation	10 kDa culture filtrate antigen cfp10, Esat-6 like protein, component of a novel secretion apparatus
13	152	esxA	esxA	present	present	esxA	virulence/detox./adaptation	6 kDA early secretory antigenic target Esat-6, component of a novel secretion apparatus, forms heterodimer with EsxB
13	153	MMAR_5460	MUL_5047	present	present	present	cell wall / cell processes	conserved hypothetical membrane protein
